# New Classification of Periodontal Diseases, the Obstacles Created and Opportunities for Growth

**DOI:** 10.3390/pathogens13121098

**Published:** 2024-12-12

**Authors:** Daniel H. Fine

**Affiliations:** Department of Oral Biology, Rutgers School of Dental Medicine, 110 Bergen, Newark, NJ 07103, USA; finedh@sdm.rutgers.edu

## 1. Gaps in the Knowledge, Opening a Forum for Discussion and Future Directions

The purpose of this Editorial is to expose the gaps in the knowledge created by a decision by the World Workshop Consensus Conference (WWCC), held in 2017, which was focused on the re-classification of periodontal diseases [1]. This newly developed classification system has had a negative impact on our ability to understand a specific form of periodontal disease [2]. This Special Issue of Pathogens focuses on the newly developed classification system and how it affects our ability to understand a specific form of periodontal disease, Localized Aggressive Periodontitis (LAgP), and the microorganism *Aggregatibacter actinomycetemcomitans*, which is intimately involved in disease initiation and progression [2]. As a result of the conclusions of the 2017 conference, research related to LAgP and *Aggregatibacter actinomycetemcomitans* has been minimized, as have the unique features of (1) LAgP, the “disease” (2) the key essential microbe related to this disease, and (3) replicated research that supports the uniqueness of both the microbe and the disease have been squelched [3,4]. This Special Issue also illustrates how important studies of *Aggregatibacter actinomycetemcomitans* in well-defined cases of LAgP have led to scientific discoveries that have extended far beyond this field of research, into areas as diverse as staphylococcal infections and lymphomas [5,6]. The editorial below will discuss (1) the developments that led to this current state of affairs, (2) the outsized influence this conference has had on this field of study, (3) the failures of this particular consensus conference, and (4) the way in which future failures can be overcome by well-designed consensus conferences.

A review of recent history will provide a better understanding of how these “new” definitions evolved and the effect these “new” definitions have had on this field of study. In the specialty of periodontology, over the last 60 years or more, there have been repeated efforts to review the current literature and update information regarding disease classification [7]. Global workshops have occurred, typically every 7 to 10 years, which were designed to review diagnosis, treatment, and prevention, with the goal of presenting the most current data that could function as a guide for practitioners, academicians, and researchers [7,8]. The direction over the past thirty to forty years has been focused on efforts to separate diseases based on their unique presentation [9]. However, the most recent workshop has taken a distinctly conservative and restrictive approach to periodontal disease and more or less minimized the value of disease classifications that distinguished between aggressive/rapidly progressive forms of disease, disease that is refractory to standard treatment, and diseases that are slowly progressive [1,10,11]. This strategy goes against the fundamental approach in the health profession that led to personalized/precision medicine, which is designed to emphasize subtle differences in diseases with an eye toward providing a more precise guide to treatment and prevention [12]. Interestingly, the relationship between periodontal disease and its effect on systemic diseases such as diabetes [13] and coronary heart disease, as well as their effects on periodontal disease, have been retained [1,11,14]. While data related to cohorts of bacteria and their relationship with the local host response and forms of periodontitis have been accumulating, this aspect has been minimized. In many cases, scientific truths take time to be accepted; however, in some cases, misguided objections to factual data can outweigh proven truths. What seems obvious to some is an anathema to others [15,16].

Research is intended to uncover scientific principles based on facts through a process of repeated testing and replication intended to support or refute the original hypothesis. However, research is conducted by humans, all of whom have foibles that can introduce prejudices and biases that can undermine the intentions and findings of the original research. The recognition that specific forms of the most common dental diseases, caries, and periodontitis are dependent on a complex imbalance in the local ecology, with overriding genetic and sociological component is logical [17,18]. The data supporting these linkages are convincing but still have missing fragments. Putting these pieces together takes time and is a common goal in several other branches of biological research; however, throwing the baby out with the bathwater is also common and an ill-conceived approach [19]. It appears as if profound experimental proof changes some opinions; however, landmark experiments occur infrequently and are usually dependent on a small group of insightful/influential researchers [20]. Max Planck developed a principle that suggests that “new scientific truth does not occur by convincing opponents but results from the death of its opponents” [15]. Simply put, scientific progress, as per Planck, occurs one funeral at a time [16]. While the direction of oral biology has been moving forward, we are facing the feeling that some of our major decisionmakers are unwilling or unable to accept the concept of personalized/precision medicine. Proof of the relevance of the specificity and importance of an accurate diagnosis can be seen in another branch of our specialty, the area of pediatric dentistry, where the diagnosis of subtypes of caries has had a major impact on the prevalence of caries in children [2]. The diagnosis of occlusal as opposed to proximal caries resulted in the use of occlusal sealants, reducing the prevalence of occlusal decay [21]. In the case of the diagnosis of early childhood caries (ECC), dietary restrictions resulted in a reduction in ECC [22]. In the most recent World Workshop on Periodontal Diseases, the “consensus” reached by a group of individuals gathered at this world workshop in 2017 reversed scientific progress. This may be explained by the death of major advocates of the microbiology and immunology of periodontal diseases, allowing the doubters to emerge as the more influential and powerful group [23,24].

One example of this cycle of discovery and lack of support took place in 1848, when Dr. Ignaz Ignaz Semmelweis, a Hungarian physician/obstetrician, observed and reported that puerperal fever at the time of birth resulted from poor antiseptic practices by the physicians who were in charge of delivery [25]. He noted that the midwives in the surgical ward he attended washed their hands prior to the delivery of pregnant mothers, whereas the physicians who had just come from teaching anatomy entered the operatory without washing their hands prior to delivery. This omission, he postulated, resulted in the much higher death rate in the babies delivered by physicians as compared to deliveries attended to by midwives [26]. He then conducted a simple set of experiments, which compared delivery after hand-washing with a chlorine rinse to delivery after no hand-washing and showed conclusively that puerperal fever was significantly reduced if those delivering the babies washed their hands thoroughly prior to delivery. His presentations to societies throughout Europe fell on deaf ears and were only accepted after Dr. Joseph Lister repeated these experiments by demanding hand-washing and spraying phenolic acid in the delivery rooms he supervised [27]. These studies were first presented in 1865 and were published in 1867. However, in 1865, Semmelweis was committed to a mental institution and died never knowing that his basic experiment was finally accepted by his medical colleagues.

This editorial and the upcoming book were designed to provide an open forum for discussion among researchers and clinicians who are opposed to the position taken at the last consensus conference. Vigorous discussion has not been encouraged, nor has there been any significant discussion related to this new classification [28]. This failure in communication needs to be corrected and is especially unacceptable in a complex scientific endeavor. As a result, an active field of research has been affected, and this failure will continue to hamper research and clinical care in a specific form of periodontitis that occurs in children and adolescents of African descent [2]. We used this Special Issue to focus on LAgP, but other forms of periodontitis need to be considered as well. The rationale for the emphasis on LAgP is that tooth loss occurs at an early age and the severity of the disease can possibly compromise the overall health of the individuals affected.

The book will describe the outsized influence the WWCC decision has had on this field of research, resulting from the failure (1) to follow best practices for consensus conferences, (2) to show an open-minded appreciation of longitudinal studies that have been replicated and support key elements demonstrating differences in this silent and orphan, or rare, “disease” as compared to other forms of periodontitis, and (3) to separate disease categories into well-defined groupings and to differentiate the subtle and unique differences between divergent forms of periodontal disease [28].

The second part of this Editorial will present a brief background of oral microbiology and how it fits into the overall scheme of microbiological discoveries that currently relate to periodontitis. The final piece of the editorial will present a very brief overview of the organization of the articles contained within the Special Issue.

## 2. A Brief Overview of the Long History of Oral Microbiology and Periodontal Disease

Oral microbiology has been intimately connected to general microbiology since its inception. For those unfamiliar with the history of microbiology and oral disease, a brief review focusing on the connection between oral microbes in the oral cavity and microbiologically induced diseases in general is provided. Antonie van Leeuwenhoek (1632–1723), originally a haberdasher from the Dutch Republic who later became a lens-maker and self-taught biologist, notably documented the microbes (he termed them “animalcules”) that he visualized in his simple microscope [29,30]. Generally regarded as the “Father of Microbiology”, Van Leeuwenhoek started examining pond water, saliva, and almost everything he could find, extensively documenting the size and shape of the things he magnified with his primitive microscope. His diagrams and descriptions of the images he visualized were published for the benefit of his scientific peers. In his words, “I…took the material…from the gums above my front teeth…I found a few living animalcules.” He hand-drew pictures of creatures including cocci, spirochetes, and fusiform bacteria. In another one of his observations, he said “I took in my mouth some very strong wine-Vinegar, and closing my teeth, I gargled and rinsed them very well… but there were an innumerable quantity of animalcules yet remaining… I took a very little wine-Vinegar and mixt it with the water…[i.e., in vitro] whereupon the Animals dyed presently…. From hence…I conclude, that the Vinegar with which I washt my Teeth, kill’d only those Animals which were on the outside… but did not pass thro the whole substance of it…the scurf” [this “scurf” is what we now refer to as a biofilm] [29,30].

Two hundred years later, Louis Pasteur (1822–1895) and Robert Koch (1843–1910) founded the fields of immunology and microbiology [31,32]. Fortunately for the emerging field of dentistry, Willoughby Miller (1853–1907), a dentist trained at the Philadelphia Dental College, traveled to Germany in 1879, stayed there for many years, and studied classical methods of microbiology in the laboratory of Koch starting in the 1890s [33]. There, he proposed that the fermentation of carbohydrates occurs in the presence of oral bacteria, resulting in the formation of acid and destruction of enamel [34]. He also proposed the “focal theory of infection”, stating that oral microbes play a role in diseases at sites distant from the oral cavity [34]. This theory was not substantiated. Miller’s career choice was influenced by his father-in-law, Dr. Alexander Abbott (1860–1935), director of the “Laboratory of Hygiene”, located next door to the Dental College, who authored the first textbooks of microbiology in the USA, called *The Hygiene of Transmissible Diseases* and Principles of Bacteriology [33]. Furthermore, Dr. David Bergey (1860–1937), a member of Abbott’s department and the creator of Bergey’s Manual of Determinative Bacteriology, was another key contributor to bacteriology in the USA [33]. A young student from England, Theodor Rosebury (1904–1976), enrolled at the University of Pennsylvania Dental School, attained his dental degree in 1928, and then was offered a fellowship at Columbia University, where he immersed himself in microbiology and began to study caries and periodontal disease from a microbiological perspective [35]. Although microbiology was a field in its embryonic stages, numerous publications related to oral microbiology and disease were starting to appear. Those further interested in this history can peruse a 1918 article by Kritchevsky and Seguin [36], a 1918 paper by Turner and Drew [37], and a 1929 report by Beckwith et al. [38]. These publications likely stimulated Rosebury in his pursuit of a career in microbiology. Over the years, Rosebury and colleagues pioneered vaccine development for caries, and developed the methodologies used to study anaerobic microorganisms [35]. Famous for his work on the anaerobic chamber and anaerobic microbiology, during WWII he was appointed the Director of Germ Warfare at Fort Detrick, Maryland [35]. Rosebury notably was a mentor of JB MacDonald (1918–2014), the first director of the Forsyth Institute (appointed in 1956), who assembled a team of researchers that included Dr Sigmund Socransky (1934–2011) and Dr. Ronald Gibbons (1932–1996), among others [35]. This very brief and limited historical review omits many researchers who made important contributions to field, such as Hemmens and Harrison (1942; [39]), Fish (1937; [40]), Box (1947; [41]), Waerhaug and Steen (1952; [42]), and Shultz Haudt et al. (1954; [43]). Toward the end of the 1950s, the question as to whether infectious diseases are caused by a single microbe such as *Bacteroides melaninogenicus* (proposed by JB MacDonald and the Forsyth group [44] or by a consortium of microbes (proposed by Rosebury and colleagues [45] was disputed by microbiologist of that era; this was a forerunner of the hypotheses that provided possible explanations regarding the causes of periodontal diseases (Loesche, 1976; [46]. This controversy more than likely stimulated Walter Loesche (1935–2012), a graduate student at MIT/Forsyth/Harvard, to propose “The Specific Plaque Hypothesis (SPH) and Non-Specific Plaque Hypotheses (NSPH)”, as discussed below (Loesche, 1976; [46]). The difficulties in growing anaerobic subgingival bacteria stimulated Socransky and his colleagues (1991; 1998; [47,48]), Wade et al. (1997; [49]) Floyd Dewhirst and Bruce Paster [50,51], who were among the forerunners of the development of methods used to enumerate the uncultivable microbes that lead to qPCR.

Over the years, several hypotheses have been presented by microbiologists, intended to guide researchers in their studies and to establish proofs of the relationships between microbes and infectious diseases. Notable among these were original concepts proposed by Friedrich Gustav Henle (1809–1885), which were later modified by Koch, who, in 1890, proposed his “postulates”, which required several steps of proof to conclude that a specific microbe causes a disease. First, these postulates proposed that the microbe of interest must be found in the disease but not seen in healthy subjects. Then, the microbe must be isolated from the diseased person and grown in pure culture. Second, once isolated in pure culture, this microbe should be placed into a healthy animal and a similar pattern of disease should emerge. Finally, the organism should be re-isolated from the experimentally induced diseased animal [31,32]. These postulates were modified many times. The postulates were especially difficult to prove when the diseases were viral in nature, or when “causative” microbes could not be isolated in pure culture. Nonetheless, these postulates provided a framework for exploration. Other obstacles occurred when the “focal theory of infection” was proposed. With reference to oral infections, this theory proposed that commensal microbes from the oral cavity that were not necessarily pathogenic in nature were suspected of causing diseases distant from the oral cavity [52]. More recently, the “Bradford Hill Criteria”, proposed in 1965, included nine proofs that ideally must be met in order to conclude that the proposed microbe is causative of the disease in question [53]. While more comprehensive than Koch’s postulates, the criteria do not emphasize the importance of host susceptibility. These criteria are discussed in more detail in this Special Issue/Book [2]. With respect to dental diseases, several important hypotheses have been proposed in efforts to understand the etiology of these diseases, which include the following: (1) The Specific Plaque Hypothesis (SPH), the Non-Specific Plaque Hypothesis (NSPH) [46], the Ecological Plaque Hypothesis (EPH) ([Marsh 1994; [54]), and the Keystone Plaque Hypothesis (KPH; Hajishengalis et al., 2012 [55]).

These hypotheses propose alternative conceptual explanations of disease initiation in efforts to guide treatment and prompt further study. The SPH suggests that a particular microbe or group of microbes are responsible for a particular form of dental disease, which can be treated by reducing the particular microbe(s) with either antibiotics or antiseptics. The NSPH suggests that the accumulation of a quantity of plaque leads to disease, which can be managed by reducing the volume of plaque or its metabolic components. The EPH states that microbial ecology is influenced by the nutrients supplied to the plaque environment, either through external factors such as (1) sugars in the case of caries, or (2) microbiologically induced growth factors, and/or (3) through the host and/or microbial factors produced in response to environmental shifts. These factors can be attributed to the results that progressing disease has on microbial succession or host response elements (Marsh, 1994 [53]). Thus, in one example, the excessive consumption of carbohydrates will cause a plummet in dental plaque pH, which will increase the likelihood of the overgrowth of acidogenic (acid-producing) microbes living within that biofilm/plaque matrix. In another example, heme and menadione, derived from red blood cells emanating from microbial-induced inflammation in gingival tissues, can provide an ecological advantage to *Porphyromonas gingivalis* through promoting its continued growth and survival thanks to the nutritional factors derived from inflamed tissue. The KPH proposes that particular low-abundance microbes such as *P. gingivalis* can act as a “Keystone” microbe that modulates the host immune system to protect itself and other members of the subgingival microflora. This host modulation can support and encourage the growth of other less adaptable microbes that are biogeographically close to the co-inhabiting *P. gingivalis* microbes and that provide a cover for these less adaptable microbes in a hostile subgingival environment (Hajishengalis et al., [55]).

Our research group used the phrase “social influencer” to capture the idea that microbes like *Aggregatibacter actinomycetemcomitans* and *P. gingivalis*, even at low numbers, can have an outsized influence on the surrounding microbes by encouraging their growth and survival [2]. We propose that the “one” can influence the “many” by suppressing the host immune system at the local level. While each of these hypotheses has contributed to our understanding of disease in the oral cavity, each has focused on the bacteria and minimized the importance of alternative ways to assess damage to the host’s periodontal tissues. As a result, we now favor “The Damage/Response Framework” presented by Casadevall and Pirofski [56]. The features of this framework that differentiate it from other microbe- or host-centric hypotheses are that (1) contributions from both the host and the microbe are required to cause virulence (a microbe-centric term), (2) the ultimate outcome of the interaction of the microbe and host is determined through the damage to the host, thus incorporating the role of the host into the concepts of virulence and pathogenicity (a more host-centric term), and (3) the concept that host damage is tied to interactions between the host and microbes that vary with time. Time-related disease can be best illustrated by two viral diseases, chicken pox [57] and polio [58]. In chicken pox, the disease appears under control but flares up in some older individuals and is manifested as shingles due to the re-emergence of the Herpes Zoster virus. In polio even though the virus is undetected, symptoms can emerge in elderly individuals due to host damage that occurred decades ago.

In the mid-1960s, dental academics’ and practitioners’ interest in the relationship between microbiology and periodontal disease was re-invigorated by the work of Keyes and Fitzgerald (1962; [59]) and Loe and colleagues (1965; [60]). However, in the year 1976, the impetus to create tools and hypotheses designed to explore the relationship between microbiology and dental disease occurred in a more systematic manner. First, two independent investigators discovered that Localized Juvenile Periodontitis (later called, LAgP) was associated with a Gram-negative capnophilic cocco-bacillus, which was named *Actinobacillus actinomycetemcomitans* at the time [61,62]. These discoveries coincided with the introduction of the Specific Plaque Hypothesis developed by Walter Loesche (1976; [46]).

**Figure 1:**
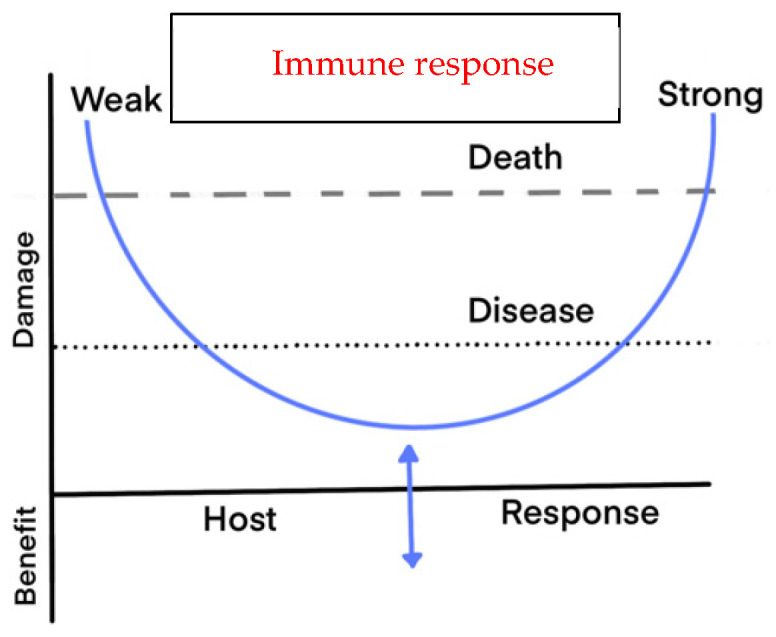
Damage/response framework. The more extreme the host response is (weaker or stronger) the worse the outcome.

As shown in Figure 1, modified from Casadevall and Pirofski [56], we propose that periodontal disease, especially localized aggressive periodontitis, is most compatible with the damage/response framework. In this case, we propose that either a weakened local host response or an over-exuberant local host response will encourage disease at the local site.

## 3. Overview of the Special Issue

The Special Issue is divided into three portions: (1) a clinical overview of the disease and the data that show its unique features; (2) the microorganism, *Aggregatibacter actinomycetemcomitans*, which is not sufficient but necessary for the disease to occur through altering the ecology enough to overwhelm the host; (3) discoveries centered around *Aggregatibacter actinomycetemcomitans* that developed as a result of intense molecular studies of this microbe. These intensive molecular investigations resulted in therapies being developed for diseases as wide-ranging as psoriasis, lymphomas, and staph infections, as well as other polymicrobial diseases, and are described in the final two papers.
